# Perceived facial age and biochemical indicators of glycemia in adult men and women

**DOI:** 10.1038/s41598-022-14555-6

**Published:** 2022-06-16

**Authors:** Agnieszka Żelaźniewicz, Judyta Nowak-Kornicka, Adriana Osochocka, Bogusław Pawłowski

**Affiliations:** Department of Human Biology, University of Wrocław, Ul. Przybyszewskiego 63, 51-148 Wrocław, Poland

**Keywords:** Predictive markers, Human behaviour, Ageing

## Abstract

Glycemia is linked with one of the key mechanisms underlying the aging process and inter-individual differences in biological age. Previous research showed that glucose level is linked with perceived age in elder individuals. This study aimed to verify if glycemia is related to perceived facial age in healthy adult individuals as interventions in younger and healthy cohorts are crucial for preventing the onset of age-related diseases. The study sample consisted of 116 healthy men of mean age 35.53 ± 3.54 years (29.95–44.29) and 163 healthy women of mean age 28.38 ± 2.40 (24.25–34.17) years. Glycemia was evaluated by fasting glucose, insulin, HOMA-IR, and glycated hemoglobin level. BMI, facial sexual dimorphism, estradiol, testosterone, and hsCRP levels were controlled. Perceived age was evaluated based on standardized facial photos in an online survey. Additionally perceived facial aging was calculated as a difference between perceived age and chronological age. No relationship between the levels of biochemical indicators of glycemia and perceived facial age or aging was found both in men and women, also when controlled for possible confounders. This study shows that perceived facial age in adult individuals is rather linked with body adiposity of sexual dimorphism but not with glycemic markers.

## Introduction

Humans age at different rates which results in major variability in inter-individual differences in physical appearance and functional capacity within the same age cohort^1^. Individuals can look younger and be healthier than might be expected from their chronological age and vice versa^2^. These differences in biological age have a strong genetic component, with heritability estimates of 27–57%, but also reflect an individual’s lifestyle, disease, and reproductive history^3,4^. Although inter-individual differences in biological age increase with age, the relationship between biological age and general condition can also be observed in younger and middle-aged adults and interventions in younger and healthy cohorts are crucial for preventing the onset of age-related diseases^5,6^.

Biological age can be assessed based on molecular (e.g. telomere length, epigenetic clock) or phenotypic biomarkers of aging, such as blood pressure, grip strength, forced expiratory volume, metabolic markers levels, cognitive or neuropsychological functioning^7–10^. However, most of these markers assess a single feature or organ function whereas different organs age at different rates (e.g. a person may be physically in good shape but not so cognitively;^11^), thus markers linked with many body functions (index derived from several biological parameters of an organism) better reflect an individual’s biological age^12^.

Perceived facial age has been shown to reliably reflect health and senesce in elders and is often used as a clinical marker of aging and predictor of mortality with a predictive value above measures of single parameters of health or cognitive ability^13,14^. Perceived age is related to various markers of biological age, such as DNA methylation [^15^; but see also Marioni et al.^16^ for negative results), leukocyte telomere length, physical and cognitive functioning^17,18^, carotid atherosclerosis^19^ and bone status in women^20^. Furthermore, perceived facial age is a marker of familial longevity in men and CVD risk in women before the onset of the disease^21^. Also, particular traits related to perceived age, such as skin wrinkling at sun-protected sites, are markers of self-assessed health and familial longevity, independently of chronological age, smoking, and BMI^22^. Although most of these studies focus on elderly individuals some studies show that these relationships might be detected in younger individuals as well^20^.

Glycemia is one of the key intrinsic factors underlying the aging process. Increased glucose level increases rates of glycation which is a spontaneous, non-enzymatic reaction between free reducing sugars, such as glucose, and free amino groups of proteins, lipids, or nucleic acids that form more stable ketamine (Amadori product). The Amadori products undergo a variety of irreversible dehydration, oxidation, polymerization, oxidative breakdowns, and rearrangement reactions that lead to the formation of early glycation end products (EGEs) and finally to advanced glycation end products (AGEs)^23,24^. AGEs are formed at lower rates by normal metabolic processes of the organism^25^, their level is partially genetically determined^26^ and many cells have developed intrinsic detoxifying pathways against the accumulation of AGEs^27^. However, glycation reaction and AGEs production may be highly accelerated in the presence of hyperglycemia, tissue oxidative stress^28^, and due to environmental factors^29^, leading to macromolecules dysfunctions, increased oxidative stress, impaired elasticity of blood vessels, skin, tendons, and faster systemic aging^30–33^.

The hypothesis that an increased glucose level acts as an “aging accelerator” has been supported by several research findings in humans and non-human animals. Offspring from long-lived families have a lower prevalence of diabetes in middle age^34,35^ and lower fasted and non-fasted glucose and insulin levels^35,36^ than controls of comparable age. Accumulation of AGEs has been detected in various tissues during aging and diabetes, including articular and skin collagen, skeletal and smooth vascular muscles or glomerular basement membranes^37–39^ and implicated in various diabetes- or age-associated pathologies of these tissues^23^. AGEs accumulation in the skin leads to impaired skin homeostasis and alteration of the balance between synthesis and degradation of the cellular matrix, modifying cells viability, gene expression, protein synthesis, and alterations, ultimately affecting also the biomechanical properties of the skin^40^, what may contribute to the older look.

Studies show that diabetes contributes to many traits typically related to skin aging, such as higher xerosis, thinner skin, longer wound healing, and compromised skin immunity^41–43^. However, so far only one study has shown that non-fasted glucose level is positively related to perceived facial age in non-diabetic elder (M_age_ = 61.8 ± 6.1 years) individuals^44,45^. Studies show that AGEs levels in children suffering from diabetes are similar to the levels in healthy adults, suggesting that AGEs may accumulate early in ontogenesis^46^, and thus the relationship between glycemia and perceived age might be detected also in young adults. Furthermore, in older individuals, this link may result from other factors, such as frailty and allostatic load or lower IGF-1 levels. As normal glycemia is crucial across ontogenesis for many components of biological condition, such as growth^47^, immunity^48^, fertility^49^ its level may be reflected in a facial appearance earlier than during post-reproductive age.

Glycemia can be measured based on several biomarkers, including markers of acute glycemia such as glucose and insulin level that may rapidly change due to an energetic state or physical effort^50^, and indicators reflecting long-term glycemia such as glycated hemoglobin level (HbA1c). HbA1c is one of the early glycation products formed by nonenzymatic glycation of hemoglobin after exposure to plasma glucose levels. HbA1c level is a genetically determined, relatively stable biomarker reflecting mean blood glucose levels during the last 8–12 weeks, commonly used in clinical practice as a gold standard for hyperglycemia screening and monitoring^51^. It is also used (in combination with an oral glucose tolerance test) for the diagnosis of diabetes and prediabetes status^52,53^. HbA1c level is associated with the risk of long-term diabetes complications and can be used for the prevention of glycation progress also in the non-diabetic patient^54,55^. Furthermore, some studies indicate that HbA1c level is linked with aging in non-diabetic healthy adults over 40 years old^56^.

The aim of this study was to verify if perceived facial age is related to glycemic markers levels in adult men and women. We hypothesized that glycemia will be positively related to perceived age both in men and women. Previous research has shown that aging trajectories can be detected as early as 20 years old^5,6^. The study was conducted on adult individuals to detect individual variance in aging in a group free of age-related disease. In contrast to the previous research markers of current (fasting insulin, glucose levels, and marker of insulin resistance) and long-term (glycated hemoglobin) glucose levels were included in the study. We hypothesized that HbA1c which reflects average blood glucose levels from the last 8–12 weeks may be a better predictor of an individual’s perceived age than levels of fasting glucose, insulin or HOMA-IR (Homeostatic Model Assessment of Insulin Resistance). As facial aging may be accelerated by many external factors we controlled for factors that are already known to associate with health and appearance, i.e. smoking^57,58^, stress^59^, and the effects of sun-exposure^60^. Furthermore, perceived facial age may be also linked with body adiposity, with a negative correlation in individuals over 40 years and positive in younger individuals^58,61^, thus we have controlled for an individual’s BMI. Perceived facial age may be also linked with the level of facial sexual dimorphism, where feminine faces are perceived as younger^62,63^ and masculine as older^64,65^, thus we controlled for facial dimorphism and sex hormone levels. We have also controlled for cortisol levels as it impacts both perceived age^45,66^ and glucose metabolism^67^. Lastly, we have controlled for chronic subclinical inflammation that is associated with insulin resistance^68^ and the accelerated aging process^69^.

## Results

### Descriptive statistics

Descriptive statistics of the variables measured in men are presented in Table 1, and women in Table 2.

**Table 1:** Descriptive statistics of the studied variables in men (N = 116).

	M	SD	Min	Max
Chronological age [years]	35.53	3.54	29.95	44.29
Perceived age [years]	35.98	4.60	26.74	47.74
Perceived aging [years]^a^	0.45	3.59	− 9.19	9.91
Insulin [mIU/L]	9.22	5.06	1.90	26.30
Glucose [mg/dL]	92.11	8.21	71.90	122.70
HbA1C [%]	5.26	0.24	4.60	5.80
HOMA-IR	2.14	1.28	0.39	7.04
BMI [cm/kg^2^]	25.76	3.34	18.72	34.75
fT [ng/dl]	14.55	5.06	4.88	28.32
Cortisol [ng/ml]	325.19	65.70	147.36	516.93
hsCRP [µg/ml]	1.14	1.22	0.01	6.49
SexDim level^b^	1.15	0.61	− 0.57	2.62

^a^Difference between perceived and chronological age.

^b^The level of sexual dimorphism.

**Table 2:** Descriptive statistics of the studied variables in women (N = 163).

	M	SD	Min	Max
Chronological age [years]	28.38	2.40	24.25	34.17
Perceived age [years]	30.55	3.94	22.88	43.66
Perceived aging [years]^a^	2.17	3.79	− 6.66	12.99
Insulin [mIU/L]	6.86	3.31	2.20	22.70
Glucose [mg/dL]	88.19	6.21	75.30	109.00
HbA1C [%]	5.02	0.21	4.40	5.50
HOMA-IR	1.51	0.78	0.46	5.27
BMI [cm/kg^2^]	22.10	3.41	16.34	35.40
E2 [pg/ml]	35.06	17.60	5.00	110.00
Cortisol [ng/ml]	254.49	86.27	89.28	468.35
hsCRP [µg/ml]	1.13	1.32	0.001	6.95
SexDim level^b^	0.16	0.56	− 1.03	1.81

^a^Difference between perceived and chronological age.

^b^The level of sexual dimorphism.

ANOVA results showed no relationship between the frequency of sun exposure on perceived facial age (F(2,113) = 1.62, *p* = 0.20) or facial aging (F(2,113) = 1.51, *p* = 0.23) in men. Similarly, the frequency of alcohol drinking was not related to perceived age (F(2,113) = 1.55, *p* = 0.22) or perceived aging (F(2,113) = 1.20, *p* = 0.31) in men. Similarly in women, there was no relationship between the frequency of alcohol drinking and perceived age (F(2,160) = 0.31, *p* = 0.73) or perceived aging (F(2,160) = 1.32, *p* = 0.27). There was also no relationship between the frequency of sunbathing and perceived age (F(2,160) = 0.08, *p* = 0.92) or perceived aging (F(2,160) = 0.02, *p* = 0.98) in women. There was also no difference in terms of perceived age (t(161) = 1.84, p = 0.07) and perceived aging (t(161) = 0.73, p = 0.46) between women who regularly use sunscreen and women who don’t. As such, we did not control for these factors in the further analyses.

### The relationship between glycemic markers and perceived age

Simple correlation analysis showed no relationship between glycemic markers levels and perceived age in men (Table 3). The positive correlation between perceived aging and HOMA-IR and insulin level was close to statistical significance (*p* = 0.07 and *p* = 0.08 respectively) (Table 3).

**Table 3:** The relationship between glycemic markers and perceived age in men (N = 116).

	Chronological age		Perceived age		Perceived aging	
	r	*p*	R	*P*	r	*P*
LOG Insulin [mIU/L]	− 0.03	0.71	0.10	0.28	0.16	0.08
LOG Glucose [mg/dL]	0.04	0.66	0.14	0.13	0.14	0.13
HbA1C [%]	0.07	0.43	0.04	0.64	− 0.02	0.86
LOG HOMA-IR	− 0.03	0.78	0.11	0.22	0.17	0.07

Men who were perceived as older than their real age and men who were perceived as younger than their real age did not differ in terms of insulin level (t(114) = − 0.48, *p* = 0.63), glucose level (t(114) =  − 1.16, *p* = 0.25), HbA1C (t(114) =  − 0.06, *p* = 0.95) or HOMA-IR level (t(114) =  − 0.44, *p* = 0.66).

Simple correlation analysis showed also no relationship between glycemic markers levels and perceived age, and perceived aging in women. There was also no relationship between glycemic markers and chronological age (Table 4).

**Table 4:** The relationship between glycemic markers and perceived age in women (N = 163).

	Chronological age		Perceived age		Perceived aging	
	r	*p*	R	*P*	r	*P*
LOG Insulin [mIU/L]	− 0.10	0.18	0.03	0.74	0.09	0.23
Glucose [mg/dL]	0.05	0.50	− 0.04	0.60	− 0.08	0.34
HbA1C [%]	− 0.01	0.98	0.01	0.89	0.02	0.90
HOMA-IR	− 0.08	0.29	0.04	0.56	0.10	0.20

Women who were perceived as older than their real age and women who were perceived as younger than their real age did not differ in terms of insulin (t(161) = 1.08, *p* = 0.28), glucose (t(161) =  − 0.19, *p* = 0.85), HbA1C (t(161) =  − 0.80, *p* = 0.42) or HOMA-IR level (t(161) = 1.44, *p* = 0.15).

### The relationship between glycemic markers and perceived age with control for potential cofounders

In men, HbA1C level did not correlate with HOMA-IR value (r = 0.10, *p* = 0.29), fasting insulin level (r = 0.11, *p* = 0.26) or glucose level (r = 0.005, *p* = 0.96). Fasting insulin and glucose level correlated positively (r = 0.50, *p* < 0.001). In women, HbA1C level was not correlated with HOMA-IR value (r = 0.09, *p* = 0.25), fasting insulin level (r = 0.05, *p* = 0.53) or glucose level (r = 0.01, *p* = 0.93). Fasting insulin and glucose level correlated positively (r = 0.27, *p* = 0.001).

None of the controlled variables were correlated with chronological age in men. Perceived age correlated negatively with cortisol levels in men. The positive correlation between BMI and perceived age was close to statistical significance (Table 5). Perceived aging correlated positively with BMI in men. The positive correlation between perceived aging and hsCRP or facial masculinity was close to statistical significance (Table 5).

**Table 5:** Correlation between chronological age, perceived age, and aging and controlled variables in men (N = 116).

	Chronological age		Perceived age		Perceived aging	
	r	*p*	R	*P*	r	*P*
BMI [kg/m^2^]	0.02	0.86	0.18	0.06	**0.21**	**0.02**
fT [pg/ml]	0.01	0.93	0.10	0.30	0.11	0.22
Cortisol [ng/ml]	− 0.16	0.09	− **0.22**	**0.02**	− 0.12	0.19
LOG hsCRP [µg/ml]	− 0.01	0.92	0.13	0.17	0.17	0.06
SexDim level	− 0.01	0.91	0.12	0.20	0.16	0.08

Significant values are in bold.

In women, chronological age correlated positively with estradiol level. Perceived age correlated positively with BMI, hsCRP. Also, more masculine faces were perceived as older (Table 6). Perceived aging was positively correlated with BMI, hsCRP, and facial sexual dimorphism (more masculine women were perceived as older than in reality). Perceived aging was also negatively related to estradiol levels (Table 6).

**Table 6:** Correlation between chronological age, perceived age, and aging and controlled variables in women (N = 163).

	Chronological age		Perceived age		Perceived aging	
	r	*p*	R	*P*	r	*P*
LOG BMI [kg/m^2^]	− 0.02	0.76	**0.22**	**0.005**	**0.24**	**0.002**
LOG E2 [pg/ml]	**0.27**	**0.001**	− 0.02	0.84	− **0.19**	**0.02**
Cortisol [ng/ml]	− 0.04	0.64	− 0.07	0.39	− 0.05	0.55
LOG hsCRP [µg/ml]	− 0.09	0.24	**0.16**	**0.04**	**0.23**	**0.004**
SexDim level	0.09	0.24	**0.23**	**0.004**	**0.18**	**0.02**

Significant values are in bold.

Multiple regression analysis showed no relationship between biomarkers of glycemia and perceived age (Table 7—Model 1) or aging (Table 7—Model 2) in men (Table 7) when controlled for possible confounders. As fT was not related to perceived age or aging we did not control for this hormone in the analyses. Perceived facial age was only negatively related to cortisol level (Table 7—Model 1), whereas perceived aging was positively related to sexual dimorphism (more masculine men were perceived as older than in reality) (Table 7—Model 2). As HOMA-IR (but not HbA1C: r = 0.08, *p* = 0.41) was positively correlated with BMI (r = 0.55, *p* < 0.001) we also conducted the similar regression excluding BMI and HbA1C level but it did not impact the relationship between HOMA-IR and perceived facial age (β = 0.09, *p* = 0.36; F(4,111) = 2.39, *p* = 0.055, *adj. r*^*2*^ = 0.05) or HOMA-IR and perceived facial aging (β = 0.14, *p* = 0.14; F(4,111) = 2.63, *p* = 0.04, *adj. r*^*2*^ = 0.05).

**Table 7:** The results of multiple regression analysis for the relationship between perceived age (Model1) or aging (Model 2) and glycemic markers, controlled for BMI, cortisol, hsCRP and facial masculinity in men (N = 116).

	β	SE(β)	t(109)	P
**Model 1: Dependent variable—perceived age: F(6, 109) = 1.70, p = 0.13, adj. r2 = 0.03**				
HbA1C [%]	0.01	0.09	0.15	0.88
HOMA-IR	0.03	0.11	0.32	0.75
BMI [kg/m^2^]	0.10	0.12	0.85	0.40
Cortisol [ng/ml]	− 0.19	0.09	− 1.97	0.052
LOG hsCRP [µg/ml]	0.04	0.10	0.44	0.66
SexDim level	0.13	0.09	1.44	0.15
**Model 2: Dependent variable—perceived aging: F(6,109) = 2.22, p = 0.048, adj. r2 = 0.06**				
HbA1C [%]	− 0.06	0.09	− 0.61	0.54
HOMA-IR	0.09	0.11	0.78	0.43
BMI [kg/m^2^]	0.11	0.12	0.95	0.34
Cortisol [ng/ml]	− 0.08	0.09	− 0.81	0.42
LOG hsCRP [µg/ml]	0.10	0.10	1.04	0.30
**SexDim level**	**0.18**	**0.09**	**2.01**	**0.046**

Significant values are in bold.

Multiple regression analysis showed no relationship between biomarkers of glycemia and perceived age (Table 8—Model 1) or aging (Table 8—Model 2) in women. As cortisol was not related either with perceived age or aging in women (Table 6) we did not include this variable as a predictor. Perceived facial age was positively correlated with BMI and facial masculinity in women (Table 8—Model 1). The negative correlation between cortisol level and perceived aging was only close to statistical significance (Table 8—Model2).

**Table 8:** The results of multiple regression analysis for the relationship between perceived age (Model1) or aging (Model 2) and glycemic markers, controlled for BMI, estradiol, hsCRP, and facial masculinity in women (N = 163).

	β	SE(β)	t(109)	*P*
**Model 1: Dependent variable—perceived age: F(6, 156) = 2.72, *p* = 0.01, *adj. r2* = 0.06**				
HbA1C [%]	0.02	0.08	0.21	0.83
HOMA-IR	− 0.08	0.09	− 0.90	0.37
**LOG BMI [kg/m**^**2**^**]**	**0.20**	**0.09**	**2.10**	**0.04**
LOG E2 [pg/ml]	0.03	0.08	0.43	0.67
LOG hsCRP [µg/ml]	0.08	0.08	0.96	0.34
**SexDim level**	**0.19**	**0.08**	**2.47**	**0.01**
**Model 2: Dependent variable—perceived aging: F(6,156) = 3.42, *p* = 0.003, *adj. r2* = 0.08**				
HbA1C [%]	− 0.02	0.08	− 0.24	0.81
HOMA-IR	0.004	0.09	0.05	0.96
LOG BMI [kg/m^2^]	0.14	0.09	1.57	0.12
LOG E2 [ng/ml]	− 0.14	0.08	− 1.87	0.06
LOG hsCRP [µg/ml]	0.14	0.08	1.72	0.09
SexDim level	0.13	0.08	1.65	0.10

Significant values are in bold.

## Discussion

We found no relationship between perceived facial age and glycemic markers neither in non-diabetic men or non-diabetic women between 24–45 years. Only a positive relationship between HOMA-IR or insulin and perceived facial aging in men was close to the statistical significance, however, these variables were unrelated when controlled for steroid hormones, BMI, hsCRP, and facial sexual dimorphism. In men, perceived age was negatively related to cortisol level and positively to BMI, although the latter was only close to the statistical significance level. Also, men who looked older than their chronological age had higher BMI and these men tended also to be more masculinized. In women, perceived age was positively related to BMI, hsCRP, and face masculinity. Also, women who looked older than their real age had higher BMI and hsCRP, lower E2 levels, and were also more masculinized.

The lack of the relationship between glycemic markers and perceived age is in line with the results of the previous study showing no relationship between glucose level and perceived age in a group of men and women between 37 to 58 years^70^. This may suggest that such a relationship may be detected only in elder individuals, exposed to elevated glycemia for a longer time, or individuals suffering from diabetes, exposed to high glucose levels as was shown in the previous study^66^. On the other hand, the heterogeneity of biological age in elderly people may result from frailty^71^ and allostatic load^72^ and not only due to the effect of glucose level and accumulating AGFs that increase with age and might contribute to the result obtained by Noordam et al.^66^. These factors are not relevant in younger and middle-aged individuals and the results of this and the previous study by Bulpitt et al.^70^ suggest that perceived age or aging are not related to glycemia in younger individuals.

This is the first study employing not only measures of acute glycemia, such as fasting glucose or insulin levels, but also a marker reflecting long-term glycemia, i.e. HbA1c. However, it is possible that the lack of the relationship between perceived age and HbA1c level results from the fact that HbA1c is not a good marker to evaluate long-term glycemia in non-diabetic patients. For instance, the results obtained by Turk et al.^73^ showed that a correlation between HbA1c level and the level of advanced glycation end products formed on hemoglobin (Hb-AEG) can only be observed in patients with poor diabetic control and relatively high Hb1Ac but not in individuals with normal glycemia. Thus, possibly in healthy individuals HbA1c level is only weakly or not related to Hb-AEG levels and might also not be related to the glycation process affecting perceived aging.

It is possible that such links may be observed only when an individual looks significantly older than in reality. A previous study showed that a physician’s assessment of perceived age has very high specificity for the detection of poor health but only when a patient looks ≥ 10 years older than his or her actual age^74^. In our sample, only a few individuals were assessed as so much younger/older than their real age which might explain the lack of the relationship between glycemia and perceived age.

The results of our study show that perceived age in adults is mainly related to BMI, the level of subclinical inflammation, and facial sexual dimorphism. Although the relationship between perceived age and sexual dimorphism may result from morphological neoteny of feminine faces^75^, both BMI and subclinical inflammation are the key factors predicting current health and the risk of many diseases, including cardio-metabolic disorders also in younger individuals^76,77^. This suggests that perceived age and aging may be also valid markers of current and future health in relatively young individuals and even relatively small differences in perceived age may help to identify individuals at risk for later age-related disorders, serving as a measure of relative fitness, and predicting disability in later life and mortality independent of chronological age^71,78^. Such studies may be especially important for early interventions in western populations, where life expectancy (the average life span of a general population) has increased in recent decades, however, the fundamental aging process remains unchanged^79^.

The shortcoming of this study is the cross-sectional study design that is prone to errors in physiological markers assessment and a possibility of inclusion of atypical for individual levels of the studied markers. However, we thoroughly controlled for any possible confounders that might impact glycemic markers levels and we have also included markers that reflect long-term glycemia (i.e. HbA1C). The cross-sectional study design also does not allow to exclude the possibility that the differences in perceived age in adults may be linked with differences in the age of onset of hyperglycemia in later life or that glycemia in younger age may predict biological age in later life, what should be verified in future longitudinal studies.

## Material and methods

The study was conducted following the tenets of the Declaration of Helsinki. All subjects were fully informed about the objective of the study and signed an informed consent form. Data used in this study were collected as a part of two broader projects. The first project concerned men’s health and included 209 participants between 26 and 45 years (M_age_ = 35.27, SD_age_ = 3.49). The research was approved by the Ethics Committee at Wrocław Medical University (nr 222/219). The second project concerned women’s health and included 211 participants (M_age_ = 28.36, SD_age_ = 2.43). The research was approved by the Ethics Committee of the Lower Silesian Chamber of Physicians (2/BO/2016).

In both studies, during the visit, a fasting blood sample was taken between 7:30 and 9:00 a.m. for further blood biochemical and hormonal analyses. Participants were weighed, measured and BMI was calculated. Photographs of faces were taken. Participants also filled out personal questionnaires, containing questions on date of birth, education level, frequency of sunbathing, and alcohol consumption, and also to verify their health status, questions on past and current health problems, and medication use.

### Participants

#### Men

A total of 209 Polish men aged 26–45 years were recruited through local media advertisements. None of the participants had any particular skin disease and there were no regular users of UVA cabins. Twenty one men were excluded due to: (a) regular smoking (N = 7); (b) inflammatory state, indicating ongoing infection—CRP level > 10 mg/dl (N = 1); (c) reported chronic diseases (N = 4); (d) incomplete data (N = 9). After this initial exclusion, we excluded men with a beard as having a beard impacted a man’s perceived age (N = 72). Thus, the final sample consisted of 116 healthy and non-smoking men of mean age 35.53 ± 3.54 years (29.95–44.29) years.

#### Women

A total of 211 Polish women aged 24–34 years were recruited through local media advertisements. None of the participants had any particular skin disease or disorder. All women were nulliparous, did not use hormonal contraception and were invited for the study visit at the same moment of the menstrual cycle (early follicular phase). From this group forty-eight women were excluded due to: (a) reported chronic disease (N = 10); (b) regular use of UVA cabins (N = 3); (c) frequent smoking (N = 9); (d) inflammatory state, indicating ongoing infection—CRP level > 10 mg/dl (N = 3). We have also excluded participants whose data were incomplete (N = 18) and were on a different day of the menstrual cycle (N = 5). Thus, the final sample consisted of 163 healthy and non-smoking women of mean age 28.38 ± 2.40 (24.25–34.17) years.

### Perceived age assessment

An en-face photograph of the face was acquired for all participants under standardized photographic conditions with a digital still camera (Nikon D7100 with Tamron SP AF 17–50 mm F/2.8 XR Di II LD IF camera lens). Camera-to-head distance and camera settings were held constant. Participants had no make-up, and were asked to have a neutral facial expression, remove glasses or earrings, and wear a hairband if needed. Photographs were standardized in terms of size based on pupil distance and an oval was placed around the face to obscure the hairstyle and color.

Photos were assessed in terms of perceived age in an online survey by assessors unaware of participants’ age. Participants were answering an open question: *“How old is the person in the photo”* and mean values were used in the analyses*.* We have also calculated an additional variable—perceived aging—that was calculated as a difference between perceived and chronological age (perceived aging = perceived age—chronological age). The higher the values the older a person looks.

#### Men’s perceived age assessment

1024 heterosexual Polish women of mean age between 18–39 years (M_age_ = 22.51 ± 3.74) took part in the study. Images were presented to them in a randomized order and each participant rated 15 photographs. The mean perceived age was generated from an average of 73.5 independent assessments of age (range 54–91 assessments).

#### Women’s perceived age assessment.

1361 heterosexual Polish men of age between 18–39 years (M_age_ = 23.54 ± 5.27). Images were presented to them in a randomized order and each participant rated 15 photographs. The mean perceived ages were generated from an average of 97.7 independent assessments of age (range 78–121 assessments).

### BMI & facial sexual dimorphism measurements

BMI was calculated from measurements of weight (in kilograms) divided by height (in meters squared).

Facial shape sexual dimorphism (facial SD) was measured in the photos. Face-shape sexual dimorphism was measured from each photograph, using a vector analysis method^80^, following methodology from Cai et al.^81^, using code for R script by Holzleitner et al. (available at https://osf.io/98qf4/; R script for analyzing sexual dimorphism scores following Scott et al.^82^ and Komori et al.^83^). A lower score indicates a more feminine face shape. An additional adult 50 male (M_age_ = 27.67 years, SD_age_ = 3.14 years) and 50 female (M_age_ = 25.92 years, SD_age_ = 1.85 years) faces (recruited from the same population) were used to build the model used to calculate sexual dimorphism scores.

### Physiological markers measurements

#### Glycemic markers

Fasting blood samples were collected during the participants’ visit to a laboratory. Participants were asked to refrain from physical activity, alcohol drinking, and heavy meals for 24 h prior to the study visit.

Glycemic markers, including fasting plasma glucose, insulin, and glycated hemoglobin (HbA1C) were assayed in a certified analytical laboratory (DIAGNOSTYKA). HOMA-IR index [calculated based on fasting glucose and insulin levels, according to the formula: insulin (mU/ml) × glucose (mmol/l)/22.5].

#### Inflammation level (hsCRP)

Serum hsCRP in men and women was measured by immunoassay and commercial ELISA kit (DEMEDITEC cat. no. DE740011). Inter- and intra-assay precision provided by the manufacturer were < 6.3%, < 6.9%. Assay sensitivity was 0.02 µg/ml. Sample and reagents preparation, as well as assay procedures, were carried out in accordance with the manufacturers’ instructions. Samples were assayed in duplicate and the average absorbance value was used to calculate hormone concentration. Standard curves were created by plotting the mean absorbance value (Y-axis) for each standard against its concentration (X-axis). The best fit line was used for calculating the individual's levels of hsCRP in each sample. The concentrations were expressed in µg/ml.

#### Hormone levels

Serum cortisol levels in men and women were measured in the Department of Human Biology at the University of Wroclaw. Competitive ELISA kits (DEMEDITEC cat no DE3388) were used for the quantitative determination of cortisol. The analytical sensitivity of the test was 3.79 ng/ml with inter- and intra-assay variations of less than 6.4% and less than 8.0% respectively. The test procedure was conducted following the manufacturer’s instructions. Serum samples with unknown cortisol levels and calibrators (with the known concentration of cortisol supplied with each kit) were assayed in duplicate and average values were used to calculate the participants’ cortisol levels. The hormone concentration values were calculated in relation to a plotted standard curve (Y-axis—standard absorbance; X-axis standard concentration). Cortisol hormone levels were expressed in ng/ml.

Serum estradiol (E2) level in women was assayed in the certified laboratory (DIAGNOSTYKA) using ElectroChemi Luminescence immunoassay and Cobas analyzer (Roche Diagnostic) and expressed in pg/ml.

Serum free testosterone (fT) in men was assayed in the certified laboratory (DIAGNOSTYKA) using the ELISA method and commercial kits (NovaTec) and expressed in ng/dl.

### Statistical analysis

The normality of the variables was assessed based on distribution graphs, and kurtosis and skewness tests. In men, the values of insulin, glucose, HOMA-IR, and hsCRP were not distributed normally across participants, thus logarithmic values were used in the analyses. In women, the values of insulin level, BMI, estradiol, and hsCRP differed from normal distribution thus, logarithmic values were used in the analyses. The distribution of logarithmized variables did not differ from the normal distribution and none of the variables had outliers exceeding the value of M ± 3SD.

As there was a difference in chronological age and average glycemic markers levels (*p* < 0.05) between men and women all the analyses were run separately for men and women.

Based on their responses to a personal questionnaire men were divided into three categories: (a) never sunbathing (N = 12), (b) only during sports practice (N = 63), (c) regular sunbathing during summer and sports practice (N = 41). According to alcohol consumption men were divided into the following categories: (a) once per month or less often (N = 34); (b) 2–3 times per month (N = 52); (c) more often than twice per week (N = 30). Based on their responses to the personal questionnaire women were divided into three categories: (a) never sunbathing (N = 22), (b) only during sports practice (N = 79), (c) regular sunbathing during summer and sports practice (N = 62). According to alcohol consumption women were divided into the following categories: (a) once per month or less often (N = 39); (b) 2–3 times per month (N = 100) (c) more often than twice per week (N = 24). We used the ANOVA test to verify if the frequency of sunbathing and alcohol use was related to perceived facial age. Women were additionally asked if they use sunscreens regularly and divided into the yes (N = 122) and the no (N = 41) groups. We compared the mean perceived age and perceived aging scores between the two groups with t-test.

We used Pearson correlation analyses to verify if perceived age, perceived aging and also chronological age are related to glycemic markers (fasting glucose and insulin levels, HbA1C, HOMA-IR). We also used a t-test to verify if individuals who were perceived as older than in reality (N_men_ = 60; N_women_ = 110) and individuals who were perceived as younger than in reality (N_men_ = 56; N_women_ = 53) differed in terms of glycemic markers.

Finally, we have run a series of regression analyses. As dependent variables, we used perceived age or perceived aging. As predictors, we introduced biomarkers of glycemia (HOMA-IR and glycated hemoglobin), testosterone level (in men)/estradiol level (in women), BMI, cortisol, and hsCRP. In regression analyses, we only used glycated hemoglobin and HOMA-IR as biomarkers of glycemia, as HOMA-IR may be a better marker of glycemia than fasting glucose or insulin levels alone. Prior to the analysis, we tested for possible correlations between predictors with Pearson correlation analysis.

Analyses were performed with Statistica 13.0 software (TIBCO Software Inc. (2017), Statistica ver. 13, http://statistica.io.). The results were interpreted as statistically significant if *p* < 0.05.

## Supplementary Information

Supplementary Information 1.

Supplementary Information 2.
